# Association Between Physical Activity Levels and Chronic Disease Risk Among Korean Adults with Sleep Deficiency

**DOI:** 10.3390/jcm14238398

**Published:** 2025-11-26

**Authors:** Jongsuk Park, Seohyung Yang, Sukyool Jung

**Affiliations:** 1 Department of Sport and Health Studies, Konkuk University, Chungju-si 27478, Republic of Korea; model200@kku.ac.kr; 2 School of Global Sport Studies, Korea University, Sejong-si 30019, Republic of Korea; trusting2000@naver.com; 3 Sports Medicine Major, Department of Healthcare Convergence, CHA University, Pocheon-si 11160, Republic of Korea

**Keywords:** physical activity, sleep deficiency, abdominal obesity, diabetes, metabolic syndrome, depression, KNHANES

## Abstract

**Background/Objectives**: This study investigated the association between physical activity (PA) levels and the risk of chronic diseases in Korean adults with sleep deficiency (SD). **Methods:** Data were obtained from the Korea National Health and Nutrition Examination Survey (2016–2021; *n* = 31,338). SD was defined as less than 7 h of sleep per night. The PA levels were categorized as low, moderate, or high. Multivariable logistic regression was used to estimate odds ratios (OR) with 95% confidence intervals (CI) for various chronic diseases, adjusting for demographic and lifestyle covariates. **Results:** High PA levels were associated with lower odds of abdominal obesity (OR = 0.855, 95% CI = 0.782–0.934, *p* < 0.001), hypertension (OR = 0.787, 95% CI = 0.657–0.942, *p* < 0.01), diabetes mellitus (OR = 0.743, 95% CI = 0.622–0.887, *p* < 0.01), and metabolic syndrome (OR = 0.706, 95% CI = 0.586–0.850, *p* < 0.001). Moderate PA showed similar but weaker associations. Conversely, high PA levels were associated with higher odds of depression (OR = 1.474, 95% CI = 1.123–1.935, *p* < 0.01). Subgroup analyses indicated that the protective effects of PA were stronger among women, non-smokers, and individuals with obesity. **Conclusions:** Among adults with SD, moderate-to-vigorous PA is associated with a lower odds of several metabolic disorders, including abdominal obesity, diabetes, and metabolic syndrome. This highlights the importance of regular PA in maintaining metabolic health. However, a positive association between high PA and depression should warrant further investigation, as reverse causality or residual confounding may explain this association.

## 1. Introduction

Sleep is a fundamental biological process that maintains various physiological and biochemical functions in the human body, including metabolic regulation, appetite control, immune function, cardiovascular and endocrine homeostasis, and emotional health [1]. Although adults are generally advised to obtain adequate sleep (7–9 h) [2], insufficient sleep has become increasingly common worldwide, with a substantial proportion of adults reporting less than the recommended duration [3,4]. Growing evidence indicates that chronic sleep deficiency (SD) is associated with adverse health outcomes, including obesity, hypertension, diabetes, and mental health disorders [5,6,7,8]. These associations highlight the importance of understanding the behavioral and lifestyle factors that may affect health status among individuals with SD.

Physical activity (PA), which includes both structured exercise and routine daily movement, has been extensively studied as a behavioral factor that correlates with metabolic and psychological health [9]. Previous studies have consistently shown that regular PA is associated with enhanced insulin sensitivity, improved body composition, favorable cardiometabolic markers, and reduced inflammation [10,11,12]. In addition, recent research indicates that PA may help mitigate the physiological and psychological risks associated with SD [4,13,14,15,16]. However, most experimental studies in this area have primarily focused on acute or total sleep deprivation, typically defined as sleep loss lasting more than 24 h. These conditions do not accurately reflect the more common experience of SD in daily life [13]. Consequently, there is a need to investigate how habitual PA under real-world conditions relates to chronic disease risk in populations with SD.

Although the beneficial effects of PA under SD conditions have been demonstrated in experimental settings, little is known about whether habitual PA accumulated during daily life is associated with chronic disease profiles among individuals with SD at the population level. Furthermore, previous studies have not evaluated how these associations differ according to demographic or behavioral characteristics of the participants. This gap highlights the need for large-scale epidemiological evidence to examine how different levels of PA relate to chronic disease profiles within populations affected by SD.

This study aimed to investigate the association between habitual PA levels and the risk of chronic diseases among Korean adults with SD, using nationally representative data from the Korea National Health and Nutrition Examination Survey (KNHANES). To achieve this aim, the study examined differences in chronic disease prevalence and key health-related indicators across PA levels. It also evaluated the associations between PA levels and the odds of various chronic diseases through multivariable logistic regression models adjusted for demographic and lifestyle factors. Furthermore, the study investigated whether these associations varied across population subgroups defined by sex, age, BMI category, smoking status, alcohol consumption, and sleep duration.

## 2. Materials and Methods

### 2.1. Study Design and Data Source

This study utilized a cross-sectional design using nationally representative data from the KNHANES, which was collected between 2016 and 2021. KNHANES uses a multistage probability sampling method to obtain health, nutrition, and lifestyle information from the Korean population. Although the original survey design incorporated complex sampling and weighting, this study was conducted without applying survey weights, as the primary objective was to examine associations rather than to produce population-level prevalence estimates.

### 2.2. Study Population

**Figure 1:**
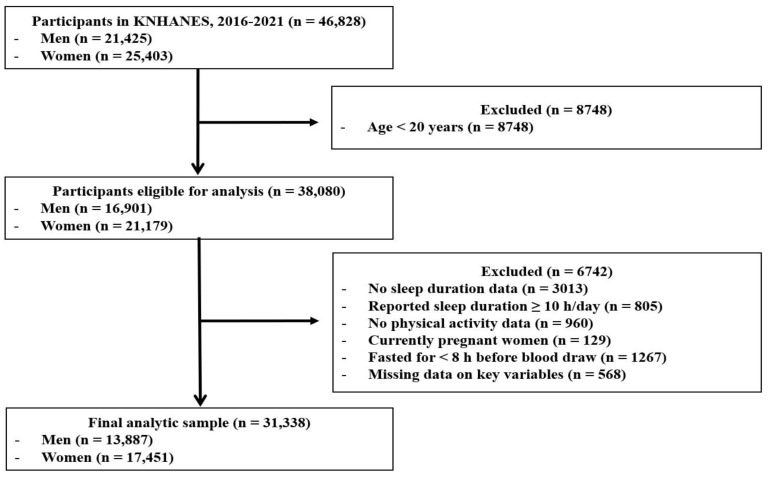
Flow diagram of participant selection from KNHANES (2016–2021). The exclusion criteria were applied sequentially. KNHANES: Korean National Health and Nutrition Examination Survey.

The raw datasets collected between 2016 and 2021 were used in this study. The initial survey population comprised 46,828 individuals (21,425 men and 25,403 women) who participated in the KNHANES during the study period. The following exclusion criteria were applied: individuals younger than 20 years of age (*n* = 8748); participants without recorded sleep duration (*n* = 3013); those reporting ≥10 h of sleep per day (*n* = 805); individuals without physical activity data (*n* = 960); currently pregnant women (*n* = 129); participants who did not meet the minimum fasting period of 8 h (*n* = 1267); and those with missing data for variables required in the analyses (*n* = 568). After applying these criteria, the final sample comprised 31,338 participants (13,887 men and 17,451 women). A flow diagram of participant selection is shown in Figure 1.

All KNHANES participants provided written informed consent according to protocols established by the Korea Disease Control and Prevention Agency (KDCPA). The survey procedures were approved by the Institutional Review Board of KDCPA (IRB No: 2018-01-03-P-A, 2018-01-03-C-A, 2018-01-03-2C-A, and 2018-01-03-3C-A). Additionally, this study was approved by the Korea University Institutional Review Board with exempt status, as it involved secondary analysis of publicly available data (IRB No: KUIRB-2025-0029-01; approved on 22 January 2025).

### 2.3. Measurement of Sleep Duration

The average sleep duration of the participants was obtained from a health interview questionnaire. Consistent with expert recommendations, SD was defined as less than 7 h per night [2]. However, sleep duration was determined based on self-reported responses collected through structured health interviews. Because this measure does not rely on objective tools such as actigraphy or polysomnography, it may be affected by recall or social desirability bias.

### 2.4. Measurement of Physical Activity Level

PA levels were assessed in metabolic equivalents of task-minutes per week (MET-min/week) using data from the KNHANES. Data were collected from the Korean version of the Global Physical Activity Questionnaire (GPAQ), developed by the World Health Organization (WHO), which has demonstrated acceptable reliability and validity [17].

The GPAQ collects information on the frequency (days per week) and duration (hours and minutes per day) of vigorous- and moderate-intensity PA across the work, leisure, and transportation domains. According to WHO guidelines, vigorous-intensity PA is defined as activities that substantially increase heart rate and induce heavy breathing, whereas moderate-intensity PA is characterized by a moderate increase in heart rate and mild shortness of breath. For each domain, minutes per day were multiplied by days per week, and metabolic equivalent (MET) values of 4.0 for moderate intensity and 8.0 for vigorous intensity were applied. Transport-related walking or cycling was classified as moderate intensity and assigned a MET value of 4.0. The totals for each domain were then summed to determine the total PA in MET minutes per week. To minimize implausible values, standard GPAQ data-cleaning procedures were implemented, including truncation of excessive durations and consistent coding decisions, as recommended by the WHO analysis guide. Each domain and total PA level were calculated according to the WHO’s GPAQ analysis protocol [17,18]. The detailed calculation formulas are presented in Table 1.

Total PA levels were categorized into three groups according to WHO recommendations and the GPAQ categorical criteria: low PA (<600 MET-min/week), moderate PA (600 to less than 3000 MET-min/week), and high PA (≥3000 MET-min/week) [17]. However, PA in this study was not measured using objective devices, such as accelerometers. Since the GPAQ relies on self-reported data, PA estimates may be subject to subjective errors or recall bias.

### 2.5. Measurement of Chronic Disease

Chronic disease status was assessed using both direct clinical measurements and self-reported physician diagnoses from the KNHANES. The chronic diseases of this study included abdominal obesity, diabetes, hypertension, dyslipidemia, metabolic syndrome, and major cardiovascular and cerebrovascular diseases (angina, myocardial infarction, and stroke). Additionally, the survey included respiratory diseases (asthma and pulmonary tuberculosis), endocrine disorders (thyroid and kidney diseases), musculoskeletal disorders (arthritis and osteoporosis), liver cirrhosis, depression, and various types of cancer (stomach, liver, colorectal, breast, cervical, and lung cancers). Abdominal obesity was defined based on the criteria established by the Korean Society for the Study of Obesity [19], whereas metabolic syndrome was diagnosed according to the guidelines of the National Cholesterol Education Program Adult Treatment Panel III (NCEP ATP III) [20]. For all other diseases, participants who answered “yes” to the physician-diagnosed variables in the KNHANES health examination survey were classified as having a chronic condition.

### 2.6. Sociodemographic and Health Behavior Variables

Sociodemographic and health behavior variables were obtained from self-reported data in the KNHANES. Sociodemographic variables included sex, age, educational level, occupation, residential area, household income, and marital status. Household income was analyzed as equivalized income quartiles provided by the KNHANES. Health behavior variables included current smoking status, alcohol consumption level, sedentary time, daily energy intake, and weekly frequency of muscle-strengthening activities. Daily energy intake was assessed using data from 24 h dietary recalls.

### 2.7. Anthropometric Measurements, Blood Pressure, and Biochemical Assessments

Anthropometric variables (height, weight, body mass index, and waist circumference), blood pressure, and fasting biochemical markers (glucose, insulin, lipid profile, liver enzymes, and HbA1c) were measured using standardized procedures conducted by trained examiners as part of the KNHANES protocol. Detailed information on the specific measurement equipment, assay methods, and laboratory instruments is provided in Supplementary File S1.

### 2.8. Statistical Analysis

All statistical analyses were performed using SPSS version 26.0 (IBM Corp., Armonk, NY, USA). Descriptive statistics were used to summarize participant characteristics. Continuous variables are presented as mean (M) ± standard deviation (SD), and categorical variables as frequencies (*n*) and percentages (%). Normality was assessed for continuous variables before parametric testing.

Initially, group comparisons between the sufficient sleep (SS) and SD groups were performed using independent samples t-tests for continuous variables and chi-square tests for categorical variables. Among participants with SD, differences across PA levels (low, moderate, and high) were evaluated using one-way analysis of variance (ANOVA) for continuous variables and chi-square tests for categorical variables. For all ANOVA tests, Bonferroni post hoc comparisons were conducted.

Logistic regression analyses were performed to estimate the association between PA groups and the risk of various chronic diseases in the SD population. Odds ratios (OR) with 95% confidence intervals (95% CI) were calculated, with PA modeled as a categorical variable (low PA group as the reference). A three-stage modeling strategy was employed. Model 1 only included the PA groups. Model 2 was further adjusted for sociodemographic variables (age, sex, educational level, household income, marital status, occupation, and region). Model 3 additionally included health behavior variables (current smoking status, average alcohol consumption, sedentary time, daily energy intake, and frequency of muscle-strengthening activities).

Subgroup analyses were performed to assess the potential effect modifications. All statistical tests were two-sided, and *p*-values less than 0.05 were considered statistically significant.

## 3. Results

### 3.1. Comparison of Sociodemographic, Clinical, and Health-Related Variables Between Sleep Groups

Significant differences were observed between the SS and SD groups across various sociodemographic, behavioral, and clinical variables. Individuals in the SD group were older and showed a higher prevalence of high-risk alcohol consumption and smoking (all *p* < 0.001). Additionally, the SD group showed significantly higher levels of anthropometric and biochemical indicators, including BMI, WC, FBG, HbA1c, HOMA-IR, TC (all *p* < 0.001), and insulin (*p* < 0.05), compared to the SS group. Blood pressure and liver enzyme levels (AST and ALT) also differed significantly between groups (all *p* < 0.01). Detailed values for all variables are presented in Table 2.

### 3.2. Differences in Sociodemographic, Health Behavior, Anthropometric, Biochemical, and Chronic Disease Variables by Physical Activity Groups in Adults with Sleep Deficiency

Among adults with SD, various sociodemographic characteristics showed significant differences across the PA groups (all *p* < 0.001). Participants with high levels of PA tended to be younger and reported lower sedentary time and greater grip strength (all *p* < 0.001). Significant group differences were also observed for anthropometric indicators, including BMI and WC, as well as biochemical markers such as TG, HDL-C, FBG, HbA1c, insulin, and HOMA-IR (all *p* < 0.05). The prevalence rates of abdominal obesity, hypertension, diabetes, dyslipidemia, and metabolic syndrome varied significantly among the PA groups (all *p* < 0.001). A detailed comparison is presented in Table 3.

### 3.3. Association Between Physical Activity Groups and Chronic Diseases Among Adults with Sleep Deficiency

In the logistic regression analyses, moderate and high levels of PA were associated with lower odds of abdominal obesity, diabetes, and metabolic syndrome in fully adjusted models (all *p* < 0.05). Additionally, high PA was associated with lower odds of hypertension. In contrast, high PA was associated with higher odds of depression (OR = 1.474, 95% CI = 1.123–1.935). Most other outcomes, such as dyslipidemia, myocardial infarction, stroke, arthritis, osteoporosis, pulmonary tuberculosis, asthma, thyroid disease, kidney disease, liver cirrhosis, and various types of cancer, did not show significant associations after full adjustment. The results for all outcomes across the three regression models are summarized in Table 4.

### 3.4. Subgroup Analyses of the Association Between Physical Activity Groups and the Risk of Chronic Diseases

Subgroup analyses demonstrated consistent associations across age, sex, BMI categories, smoking status, and alcohol consumption. Significant interaction effects were observed for abdominal obesity (sex, smoking status, and BMI), diabetes (BMI), dyslipidemia (BMI), hypertension (sex, alcohol consumption, and BMI), metabolic syndrome (sex, sleep duration, and BMI), and depression (sleep duration). The full subgroup results are presented in Table 5, Table 6, Table 7, Table 8, Table 9 and Table 10.

## 4. Discussion

### 4.1. Principal Findings and Interpretation

This study identified the association between PA levels and the risk of multiple chronic diseases in Korean adults with SD using a large, nationally representative data.

Overall, individuals with SD exhibited less favorable metabolic and clinical profiles compared with those with SS.

SD has been mechanistically linked to impaired glucose regulation, insulin resistance, increased appetite, and a higher risk of obesity [21,22,23,24]. At the hormonal level, SD alters the level of leptin and ghrelin, which leads to hyperphagia and positive energy balance [25,26,27].

Among adults with SD, higher levels of habitual PA were associated with lower odds of abdominal obesity, diabetes, hypertension, and metabolic syndrome. These results are consistent with previous epidemiological studies that show inverse associations between PA and cardiometabolic risk [10,11]. Additionally, longitudinal studies have similarly reported that regular PA can reduce the risk of type 2 diabetes and metabolic syndrome in individuals who experience chronic sleep restriction [28,29].

While the observed associations align with established evidence, our cross-sectional data cannot directly infer the specific biological pathways underlying these patterns. Prior experimental studies have suggested several potential explanations, such as improved insulin sensitivity, reduced inflammation, and enhanced metabolic signaling (e.g., glucose transporter type 4 translocation and AMP-activated protein kinase activation), which may help explain the potential interaction between SD and PA [4,30,31]. However, these mechanisms should be interpreted as hypothesized biological pathways rather than those confirmed by our results. Therefore, in this study, mechanistic descriptions are best understood as theoretical frameworks that contextualize the observed associations rather than as evidence derived from the current data.

Subgroup analyses indicated significant effect modifications in the study. The associations between PA and abdominal obesity differed by sex, smoking status, and BMI. In contrast, diabetes, dyslipidemia, hypertension, and metabolic syndrome exhibited distinct patterns of effect modification across BMI categories, sex, alcohol consumption, and sleep duration. These findings are consistent with previous studies highlighting sex differences in exercise-induced metabolic adaptations and stronger PA-related metabolic benefits among individuals with obesity or elevated adiposity levels [32,33]. Collectively, these results suggest that lifestyle behaviors and metabolic health may interact differently depending on demographic and clinical characteristics. This heterogeneity illustrates the importance of personalized or stratified approaches in future epidemiological and intervention studies.

Although SD activates sympathetic tone and disrupts BP regulation [34,35], our results did not identify consistent independent associations between PA and hypertension after multivariable adjustment. This finding is consistent with recent studies suggesting that sociodemographic and behavioral confounders, including chronotype, BMI, and job-related stress, may obscure the direct associations between PA and BP among individuals with SD [36]. Similarly, the associations between PA and dyslipidemia diminished after adjustment, consistent with existing findings indicating that lipid responses to exercise differ depending on sleep duration, dietary intake, and other lifestyle factors [37,38,39].

Descriptive comparisons indicated a lower prevalence of arthritis, osteoporosis, and cardiovascular disease among individuals with higher PA levels. However, these associations were not statistically significant in fully adjusted models. This discrepancy may be attributed to the limitations of self-reported PA measurements, the cross-sectional study design, or the possibility that certain musculoskeletal or cardiovascular benefits require long-term behavioral consistency to become evident.

In addition, no significant associations were found between PA and various cancers, thyroid disease, kidney disease, or respiratory diseases. These null results are consistent with prior epidemiological evidence indicating that many protective effects of PA may operate through long-term exposure or latency mechanisms that are not captured in cross-sectional studies [40].

A notable finding of this study was the positive association between high levels of PA and depression. Moderate PA generally serves as a protective effect against depressive symptoms [41,42]. However, excessive exercise under SD conditions may exacerbate stress, increase hypothalamic–pituitary–adrenal (HPA) axis activation, raise cortisol, and reduce neurotrophic signaling, thereby impairing mood regulation [13,43]. Overtraining combined with SD can elevate cortisol levels and disrupt mood regulation, potentially explaining this paradoxical finding [13]. Another plausible explanation is reverse causality, whereby individuals experiencing stress or depressive symptoms engage in high-intensity PA as a coping strategy. Given the cross-sectional study design, the temporal direction of this association could not be established. Future longitudinal or experimental studies are required to clarify whether high-intensity PA contributes to or results from depressive symptoms in adults with SD. 

### 4.2. Strengths of the Study

This study has several strengths, including the use of a large, nationally representative dataset and extensive adjustment for sociodemographic and behavioral covariates. Additionally, the inclusion of subgroup analyses provides more nuanced insights into effect modification across demographic and clinical characteristics.

### 4.3. Limitations of the Study

Several limitations must be acknowledged. First, all key variables, including sleep duration, PA level, and lifestyle behaviors, were self-reported, which introduces potential recall bias and subjective misclassification. Although the GPAQ has demonstrated acceptable reliability, objective measures such as accelerometry were not available in this dataset. Second, the cross-sectional design precludes causal inferences. Thus, the observed associations should be interpreted descriptively rather than as evidence of risk reduction or preventive effects. Third, this study did not apply sampling weights for the KNHANES complex design. Although unweighted analyses are used in several prior studies, the absence of weighting limits the population-level generalizability of the findings, though it does not affect the internal validity of the association estimates. Consequently, the estimates presented should be interpreted as reflecting associations within the analytic sample rather than population-level prevalence estimates. While the lack of weighting may limit the generalizability of the findings to the entire Korean adult population, it does not affect the internal validity of the associations examined in the multivariable models. Future longitudinal and interventional studies are needed to better understand the causal pathways and to determine whether modifying PA levels can meaningfully influence metabolic or psychological outcomes in this population. Additionally, future studies using weighted analyses would be valuable to confirm the population-representative magnitude of these associations.

## 5. Conclusions

In Korean adults with SD, the study identified associations between higher levels of PA and lower odds of abdominal obesity, diabetes mellitus, hypertension, and metabolic syndrome. These patterns were generally consistent across various demographic and behavioral subgroups. However, high PA was also associated with increased odds of depression, a finding that contrasts with the overall trends and requires further investigation.

Overall, these results suggest that increasing PA may be associated with more favorable health profiles among individuals with SD. However, due to the cross-sectional design of this study, the observed associations cannot establish temporal or causal relationships. Future longitudinal or interventional studies are needed to clarify the directionality of these associations and to further investigate the role of PA in individuals with SD.

## Figures and Tables

**Table 1:** Calculation of physical activity levels (MET-min/week) based on GPAQ guidelines.

Domain	Intensity	Calculation of Physical Activity (MET-min/Week)
Transport-related activity	-	4.0 × duration per week (minutes) × frequency (days/week)
Work-related activity	Moderate	4.0 × duration per week (minutes) × frequency (days/week)
Work-related activity	Vigorous	8.0 × duration per week (minutes) × frequency (days/week)
Leisure-time activity	Moderate	4.0 × duration per week (minutes) × frequency (days/week)
Leisure-time activity	Vigorous	8.0 × duration per week (minutes) × frequency (days/week)
Total physical activity	-	Sum of transport-related, work-related, and leisure-time physical activity

**Table 2:** Differences in participant characteristics, biomarkers, and disease prevalence by sleep groups.

Variable	SS (*n* = 18,304)	SD (*n* = 13,034)	*p*-Values
Sex, *n* (%)			0.220
Male	8058 (44.0)	5829 (44.7)	
Female	10,246 (56.0)	7205 (55.3)	
Age (years)	50.89 ± 16.95	51.89 ± 16.12	<0.001
Education level, *n* (%)			
≤High school	9724 (53.2)	7042 (54.1)	0.001
College graduate	7588 (41.5)	5186 (39.8)	
Graduate school graduate	978 (5.3)	794 (6.1)	
Occupation, *n* (%)			<0.001
Managers and professionals	2582 (14.1)	1965 (15.1)	
Office and administrative workers	2038 (11.1)	1429 (11.0)	
Sales workers	2405 (13.1)	1764 (13.5)	
Skilled manual and technical workers	3345 (18.3)	2634 (20.2)	
Manual laborers and agricultural/fishery workers	788 (4.3)	433 (3.3)	
Unemployed or not in the labor force	7132 (39.0)	4795 (36.8)	
Residential area, *n* (%)			<0.001
Urban	14,656 (80.1)	10,732 (82.3)	
Rural	3648 (19.9)	2302 (17.7)	
Marital status, *n* (%)			0.002
Married	14,897 (81.4)	10,784 (82.7)	
Not married	3406 (18.6)	2250 (17.3)	
Household income, *n* (%)			0.093
Low	4421 (24.0)	3084 (23.7)	
Lower-middle	4608 (25.2)	3155 (24.3)	
Upper-middle	4603 (25.2)	3372 (26.0)	
High	4627 (25.6)	3378 (26.0)	
Current smoking status, *n* (%)			<0.001
Current smoker	3019 (16.5)	2362 (18.1)	
Never smoker	15,257 (83.5)	10,659 (81.9)	
Alcohol consumption level, *n* (%)			<0.001
Never drinker	8422 (46.1)	6067 (46.6)	
Low-risk drinker	7187 (39.3)	4875 (37.4)	
Middle-risk drinker	1481 (8.1)	1109 (8.5)	
High-risk drinker	1192 (6.5)	971 (7.5)	
Level of Physical activity, *n* (%)			<0.001
Low	10,531 (57.5)	7229 (55.5)	
Moderate	6744 (36.8)	4928 (37.8)	
High	1029 (5.6)	877 (6.7)	
Frequency of muscle-strengthening activity (times/week), *n* (%)			0.003
None	13,831 (75.6)	9642 (74.0)	
1–2 Days	1548 (8.5)	1136 (8.7)	
≥3 Days	2920 (16.0)	2255 (17.3)	
Grip strength (kg)	27.99 ± 9.88	28.62 ± 9.96	<0.001
Sedentary time (hours/day)	8.08 ± 3.39	8.77 ± 3.85	<0.001
Daily energy intake (kcal/day)	1894.94 ± 831.94	1890.29 ± 888.39	0.666
Height (cm)	163.56 ± 9.19	163.45 ± 9.43	0.312
Weight (kg)	64.08 ± 12.53	65.12 ± 12.99	<0.001
BMI (kg/m^2^)	23.86 ± 3.55	24.26 ± 3.63	<0.001
WC (cm)	82.76 ± 10.37	83.97 ± 10.49	<0.001
TG (mg/dL)	131.06 ± 108.57	133.21 ± 104.64	0.079
TC (mg/dL)	190.86 ± 37.96	192.72 ± 38.21	<0.001
LDL-C (mg/dL)	113.08 ± 35.27	114.10 ± 35.34	0.012
HDL-C (mg/dL)	51.57 ± 12.58	51.98 ± 12.96	0.005
FBG (mg/dL)	100.73 ± 22.23	102.03 ± 23.57	<0.001
HbA1c (%)	5.73 ± 0.79	5.80 ± 0.84	<0.001
Insulin (μU/mL)	8.86 ± 6.73	9.12 ± 7.52	0.023
HOMA-IR	2.28 ± 2.04	2.41 ± 2.72	<0.001
SBP (mmHg)	118.84 ± 16.49	119.56 ± 16.43	<0.001
DBP (mmHg)	75.13 ± 9.93	75.83 ± 9.98	<0.001
AST (IU/L)	23.82 ± 14.50	24.28 ± 13.39	0.004
ALT (IU/L)	22.58 ± 18.21	23.53 ± 18.47	<0.001
Prevalence of metabolic disorders, *n* (%)			
Abdominal obesity	5696 (31.1)	4730 (36.3)	<0.001
Hypertension	7480 (40.9)	5621 (43.1)	<0.001
Diabetes mellitus	6864 (37.5)	5221 (40.1)	<0.001
Dyslipidemia	8118 (44.4)	6087 (46.7)	<0.001
Metabolic syndrome	5916 (32.3)	4644 (35.6)	<0.001
Prevalence of cardiovascular and cerebrovascular disease, *n* (%)			
Myocardial infarction or angina	525 (2.9)	402 (3.1)	0.266
Stroke	398 (2.2)	279 (2.1)	0.839
Prevalence of musculoskeletal disorders, *n* (%)			
Arthritis	2193 (12.0)	1889 (14.5)	<0.001
Osteoporosis	1431 (7.8)	1106 (8.5)	0.033
Prevalence of respiratory diseases, *n* (%)			
Pulmonary tuberculosis	644 (3.5)	523 (4.0)	0.023
Asthma	508 (2.8)	445 (3.4)	0.001
Prevalence of endocrine and renal diseases, *n* (%)			
Thyroid disease	750 (4.1)	544 (4.2)	0.738
Kidney disease	112 (0.6)	103 (0.8)	0.059
Prevalence of other diseases, *n* (%)			
Liver cirrhosis	59 (0.3)	46 (0.4)	0.644
Depression	799 (4.4)	635 (4.9)	0.034
Prevalence of cancer, *n* (%)			
Stomach cancer	145 (0.8)	91 (0.7)	0.343
Liver cancer	26 (0.1)	16 (0.1)	0.645
Colorectal cancer	106 (0.6)	70 (0.5)	0.623
Breast cancer	146 (0.8)	84 (0.6)	0.117
Cervical cancer	91 (0.5)	57 (0.4)	0.446
Lung cancer	34 (0.2)	30 (0.2)	0.391

Data are presented as mean ± standard deviation for continuous variables and as numbers (percentages) for categorical variables. Group comparisons were performed using independent sample t-tests for continuous variables and chi-square tests for categorical variables. SS, sufficient sleep group; SD, sleep deficiency group; BMI, body mass index; WC, waist circumference; TG, triglycerides; TC, total cholesterol; LDL-C, low-density lipoprotein cholesterol; HDL-C, high-density lipoprotein cholesterol; FBG, fasting blood glucose; HbA1c, glycated hemoglobin; SBP, systolic blood pressure; DBP, diastolic blood pressure; HOMA-IR, homeostasis model assessment of insulin resistance; AST, aspartate aminotransferase; ALT, alanine aminotransferase. Statistical significance was set at *p* < 0.05.

**Table 3:** Comparison of sociodemographic, health behavior, anthropometric, biochemical, and chronic disease variables by physical activity group in adults with sleep deficiency.

Variable	Low (a) (*n* = 7229)	Moderate (b) (*n* = 4928)	High (c) (*n* = 877)	*p*-Value	Post Hoc
Sex, *n* (%)				<0.001	
Male	3018 (51.8)	2270 (38.9)	541 (9.3)		
Female	4211 (58.4)	2658 (36.9)	336 (4.7)		
Age (years)	54.65 ± 15.74	49.00 ± 15.93	45.32 ± 15.55	<0.001	c < b < a
Education level, *n* (%)				<0.001	
≤High school	4388 (60.8)	2286 (46.4)	368 (42.0)		
College graduate	2461 (34.1)	2269 (46.1)	456 (52.0)		
Graduate school graduate	373 (5.2)	368 (7.5)	53 (6.0)		
Occupation, *n* (%)				<0.001	
Managers and professionals	966 (13.4)	854 (17.4)	145 (16.5)		
Office and administrative workers	727 (10.1)	610 (12.4)	92 (10.5)		
Sales workers	938 (13.0)	704 (14.3)	122 (13.9)		
Skilled manual and technical workers	1480 (20.5)	946 (19.2)	208 (23.7)		
Manual laborers and agricultural/fishery workers	286 (4.0)	100 (2.0)	47 (5.4)		
Unemployed or not in the labor force	2825 (39.1)	1707 (34.7)	263 (30.0)		
Residential area, *n* (%)				<0.001	
Urban	5690 (78.7)	4330 (87.9)	712 (81.2)		
Rural	1539 (21.3)	598 (12.1)	165 (18.8)		
Marital status, *n* (%)				<0.001	
Married	6313 (87.3)	3828 (77.7)	643 (73.3)		
Not married	916 (12.7)	1100 (22.3)	234 (26.7)		
Household income, *n* (%)				<0.001	
Low	1821 (25.3)	1077 (21.9)	186 (21.2)		
Lower-middle	1806 (25.1)	1135 (23.1)	214 (24.4)		
Upper-middle	1799 (25.0)	1347 (27.4)	226 (25.8)		
High	1767 (24.6)	1360 (27.6)	251 (28.6)		
Current smoking status, *n* (%)				<.001	
Current smoker	1337 (18.5)	811 (16.5)	214 (24.4)		
Never smoker	5883 (81.5)	4114 (83.5)	662 (75.6)		
Alcohol consumption, *n* (%)				<0.001	
Never drinker	3638 (50.4)	2112 (42.9)	317 (36.2)		
Low-risk drinker	2508 (34.7)	1999 (40.6)	368 (42.0)		
Middle-risk drinker	569 (7.9)	454 (9.2)	86 (9.8)		
High-risk drinker	506 (7.0)	360 (7.3)	105 (12.0)		
Frequency of muscle-strengthening activity (times/week), *n* (%)				< 0.001	
None	5939 (82.2)	3230 (65.5)	473 (53.9)		
1–2 Days	478 (6.6)	571 (11.6)	87 (9.9)		
≥3 Days	811 (11.2)	1127 (22.9)	317 (36.1)		
Grip strength (kg)	27.44 ± 9.88	29.53 ± 9.75	32.91 ± 10.07	<0.001	a < b < c
Sedentary time (hours/day)	9.14 ± 3.96	8.51 ± 3.65	7.24 ± 3.50	<0.001	c < b < a
Daily energy intake (kcal/day)	1818.48 ± 847.48	1946.75 ± 888.03	2173.22 ± 1117.79	<0.001	a < b < c
Height (cm)	162.25 ± 9.40	164.43 ± 9.20	167.82 ± 9.09	<0.001	a < b < c
Weight (kg)	64.11 ± 12.59	65.74 ± 13.04	69.93 ± 14.57	<0.001	a < b < c
BMI (kg/m^2^)	24.26 ± 3.65	24.19 ± 3.57	24.66 ± 3.84	0.002	b, a < c
WC (cm)	84.45 ± 10.56	83.24 ± 10.29	84.13 ± 10.81	<0.001	b < c, a
TG (mg/dL)	136.46 ± 104.58	129.60 ± 104.72	126.74 ± 103.91	<0.001	c, b < a
TC (mg/dL)	192.31 ± 39.05	193.26 ± 36.92	193.12 ± 38.39	0.385	
LDL-C (mg/dL)	113.80 ± 35.69	114.49 ± 34.69	114.33 ± 35.99	0.560	
HDL-C (mg/dL)	51.22 ± 12.88	52.85 ± 13.01	53.44 ± 12.99	<0.001	a < b, c
FBG (mg/dL)	103.30 ± 24.41	100.65 ± 22.58	99.31 ± 21.18	<0.001	c, b < a
HbA1c (%)	5.85 ± 0.86	5.74 ± 0.83	5.66 ± 0.73	<0.001	c < b < a
Insulin (μU/mL)	9.34 ± 7.68	8.90 ± 7.37	8.49 ± 6.84	0.015	c, b < a
HOMA-IR	2.48 ± 2.46	2.35 ± 3.08	2.19 ± 2.61	0.037	c, b < a
SBP (mmHg)	120.55 ± 16.90	118.39 ± 15.80	117.99 ± 15.45	<0.001	c, b < a
DBP (mmHg)	75.65 ± 10.10	76.07 ± 9.82	76.48 ± 9.81	0.011	a < c
AST (IU/L)	24.31 ± 13.71	23.97 ± 12.35	25.82 ± 16.05	<0.001	b, a < c
ALT (IU/L)	23.39 ± 17.59	23.30 ± 18.78	25.94 ± 23.11	0.004	b, a < c
Prevalence of metabolic disorders, *n* (%)					
Abdominal obesity	2834 (39.2)	1611 (32.7)	285 (32.5)	<0.001	
Hypertension	2778 (38.4)	1475 (29.9)	235 (26.8)	<0.001	
Diabetes mellitus	1112 (15.4)	569 (11.5)	80 (9.1)	<0.001	
Dyslipidemia	3530 (48.8)	2198 (44.6)	359 (40.9)	<0.001	
Metabolic syndrome	2855 (39.5)	1560 (31.7)	229 (26.1)	<0.001	
Prevalence of cardiovascular and cerebrovascular disease, *n* (%)					
Myocardial infarction or angina	260 (3.6)	121 (2.5)	21 (2.4)	<0.001	
Stroke	190 (2.6)	75 (1.5)	14 (1.6)	<0.001	
Prevalence of musculoskeletal disorders, *n* (%)					
Arthritis	1237 (17.1)	573 (11.6)	79 (9.0)	<0.001	
Osteoporosis	740 (10.2)	328 (6.7)	38 (4.3)	<0.001	
Prevalence of respiratory diseases, *n* (%)					
Pulmonary tuberculosis	320 (4.4)	175 (3.6)	28 (3.2)	0.024	
Asthma	247 (3.4)	174 (3.5)	24 (2.7)	0.491	
Prevalence of endocrine and renal diseases, *n* (%)					
Thyroid disease	279 (3.9)	234 (4.7)	31 (3.5)	0.034	
Kidney disease	55 (0.8)	38 (0.8)	10 (1.1)	0.479	
Prevalence of other diseases, *n* (%)					
Liver cirrhosis	29 (0.4)	14 (0.4)	3 (0.3)	0.564	
Depression	364 (5.0)	227 (4.6)	44 (5.0)	0.547	
Prevalence of cancer, *n* (%)					
Stomach cancer	55 (0.8)	32 (0.6)	4 (0.5)	0.517	
Liver cancer	8 (0.1)	7 (0.1)	1 (0.1)	0.886	
Colorectal cancer	41 (0.6)	25 (0.5)	4 (0.5)	0.856	
Breast cancer	53 (0.7)	28 (0.6)	3 (0.3)	0.274	
Cervical cancer	38 (0.5)	17 (0.3)	2 (0.2)	0.208	
Lung cancer	15 (0.2)	13 (0.3)	2 (0.2)	0.817	

Data are presented as mean ± standard deviation for continuous variables and as numbers (percentages) for categorical variables. Between-group comparisons were performed using one-way analysis of variance for continuous variables and chi-square tests for categorical variables. Post hoc analyses were performed using Bonferroni’s method for continuous variables. BMI, body mass index; WC, waist circumference; TG, triglycerides; TC, total cholesterol; LDL-C, low-density lipoprotein cholesterol; HDL-C, high-density lipoprotein cholesterol; FBG, fasting blood glucose; HbA1c, glycated hemoglobin; SBP, systolic blood pressure; DBP, diastolic blood pressure; HOMA-IR, homeostasis model assessment of insulin resistance; AST, aspartate aminotransferase; ALT, alanine aminotransferase. Statistical significance was set at *p* < 0.05.

**Table 4:** Multivariable logistic regression of the associations between physical activity groups and the risk of chronic diseases in adults with sleep deficiency.

Disease	PA Group	Model 1 OR (95% CI)	Model 2 OR (95% CI)	Model 3 OR (95% CI)
Abdominal obesity	Low	Ref	Ref	Ref
	Moderate	0.753 (0.698–0.813) ***	0.854 (0.789–0.924) ***	0.827 (0.759–0.902) ***
	High	0.747 (0.643–0.867) ***	0.852 (0.766–0.948) **	0.855 (0.782–0.934) ***
Hypertension	Low	Ref	Ref	Ref
	Moderate	0.684 (0.634–0.739) ***	0.941 (0.867–1.022)	0.923 (0.843–1.010)
	High	0.586 (0.501–0.686) ***	0.815 (0.693–0.959) *	0.787 (0.657–0.942) **
Diabetes Mellitus	Low	Ref	Ref	Ref
	Moderate	0.718 (0.644–0.800) ***	0.917 (0.869–0.966) **	0.910 (0.834–0.995) **
	High	0.552 (0.435–0.701) ***	0.803 (0.684–0.943) **	0.743 (0.622–0.887) **
Dyslipidemia	Low	Ref	Ref	Ref
	Moderate	0.844 (0.785–0.907) ***	0.991 (0.918–1.071)	1.002 (0.921–1.091)
	High	0.726 (0.630–0.837) ***	0.896 (0.771–1.042)	0.899 (0.762–1.062)
Metabolic syndrome	Low	Ref	Ref	Ref
	Moderate	0.710 (0.657–0.766) ***	0.873 (0.804–0.948) ***	0.867 (0.792–0.949) **
	High	0.541 (0.462–0.634) ***	0.709 (0.598–0.839) ***	0.706 (0.586–0.850) ***
Myocardial infarction or angina	Low	Ref	Ref	Ref
	Moderate	0.675 (0.542–0.840) ***	0.879 (0.700–1.103)	0.909 (0.711–1.161)
	High	0.658 (0.419–1.032)	0.995 (0.623–1.590)	1.145 (0.704–1.862)
Stroke	Low	Ref	Ref	Ref
	Moderate	0.573 (0.437–0.750) ***	0.794 (0.602–1.048)	0.839 (0.626–1.123)
	High	0.601 (0.348–1.039)	0.997 (0.568–1.749)	1.192 (0.674–2.109)
Arthritis	Low	Ref	Ref	Ref
	Moderate	0.637 (0.573–0.709) ***	0.937 (0.830–1.058)	0.918 (0.807–1.046)
	High	0.480 (0.378–0.609) ***	1.116 (0.853–1.460)	1.078 (0.805–1.443)
Osteoporosis	Low	Ref	Ref	Ref
	Moderate	0.588 (0.537–0.644) ***	0.978 (0.838–1.141)	1.043 (0.886–1.228)
	High	0.390 (0.309–0.491) ***	1.133 (0.778–1.652)	1.315 (0.888–1.946)
Pulmonary tuberculosis	Low	Ref	Ref	Ref
	Moderate	0.795 (0.659–0.959) **	0.904 (0.746–1.097)	0.950 (0.771–1.171)
	High	0.712 (0.481–1.055)	0.838 (0.561–1.251)	0.986 (0.652–1.491)
Asthma	Low	Ref	Ref	Ref
	Moderate	1.035 (0.849–1.260)	1.094 (0.892–1.342)	1.082 (0.868–1.349)
	High	0.795 (0.520–1.217)	0.908 (0.589–1.400)	0.979 (0.622–1.543)
Thyroid disease	Low	Ref	Ref	Ref
	Moderate	1.087 (0.968–1.220)	1.247 (0.902–1.725)	1.252 (0.833–1.883)
	High	0.913 (0.626–1.332)	1.375 (0.933–2.027)	1.485 (0.984–2.241)
Kidney Disease	Low	Ref	Ref	Ref
	Moderate	1.014 (0.669–1.535)	1.224 (0.799–1.875)	1.108 (0.688–1.787)
	High	1.504 (0.764–2.962)	1.859 (0.793–4.356)	2.078 (0.957–4.515)
Liver Cirrhosis	Low	Ref	Ref	Ref
	Moderate	0.707 (0.373–1.340)	0.900 (0.468–1.730)	0.859 (0.427–1.727)
	High	0.852 (0.259–2.803)	1.118 (0.333–3.752)	1.246 (0.367–4.233)
Depression	Low	Ref	Ref	Ref
	Moderate	0.911 (0.768–1.079)	1.081 (0.906–1.289)	1.092 (0.904–1.318)
	High	0.996 (0.723–1.373)	1.183 (0.880–1.591)	1.474 (1.123–1.935) **
Stomach cancer	Low	Ref	Ref	Ref
	Moderate	0.853 (0.551–1.320)	1.122 (0.718–1.752)	1.077 (0.666–1.742)
	High	0.598 (0.216–1.653)	0.916 (0.327–2.564)	1.029 (0.364–2.912)
Liver cancer	Low	Ref	Ref	Ref
	Moderate	1.284 (0.465–3.543)	1.463 (0.520–4.119)	2.015 (0.562–7.219)
	High	1.030 (0.129–8.248)	1.339 (0.163–10.978)	1.844 (0.204–16.666)
Colorectal cancer	Low	Ref	Ref	Ref
	Moderate	0.894 (0.543–1.472)	1.165 (0.700–1.940)	1.159 (0.678–1.981)
	High	0.803 (0.287–2.248)	1.102 (0.388–3.131)	1.107 (0.383–3.197)
Breast cancer	Low	Ref	Ref	Ref
	Moderate	0.774 (0.489–1.225)	0.929 (0.581–1.487)	0.911 (0.549–1.514)
	High	0.465 (0.145–1.490)	0.898 (0.276–2.921)	1.045 (0.319–3.432)
Cervical cancer	Low	Ref	Ref	Ref
	Moderate	0.655 (0.369–1.162)	0.783 (0.437–1.402)	0.843 (0.450–1.576)
	High	0.433 (0.104–1.796)	0.772 (0.182–3.266)	0.945 (0.221–4.045)
Lung cancer	Low	Ref	Ref	Ref
	Moderate	1.272 (0.605–2.676)	1.779 (0.832–3.802)	1.408 (0.569–3.485)
	High	1.099 (0.251–4.815)	1.749 (0.390–7.841)	2.041 (0.438–9.510)

Values represent odds ratios (OR) and 95% confidence intervals (CI) calculated using logistic regression. Model 1 was adjusted for the physical activity group only; Model 2 was additionally adjusted for sociodemographic variables (sex, age, residential area, household income, marital status, educational level, and occupation); and Model 3 was further adjusted for health behaviors (current smoking status, average alcohol consumption, sedentary time, daily energy intake, and frequency of muscle-strengthening activity). The low physical activity group served as the reference category for each disease. OR, odds ratio; CI, confidence interval; PA, physical activity; Ref, reference group. * *p* < 0.05, ** *p* < 0.01, *** *p* < 0.001.

**Table 5:** Subgroup analysis of abdominal obesity according to physical activity group among adults with sleep deficiency.

Subgroup	Low PA OR (95% CI)	Moderate PA OR (95% CI)	High PA OR (95% CI)	*p* for Interaction
Sex				<0.001
Men	1.0	0.881 (0.775–0.999) *	0.887 (0.716–1.097)	
Women	1.0	0.768 (0.680–0.868) ***	0.781 (0.586–1.043)	
Age (years)				0.219
20~39	1.0	0.884 (0.721–1.084)	0.998 (0.728–1.369)	
40~59	1.0	0.811 (0.708–0.929) **	0.809 (0.624–1.048)	
60~64	1.0	0.835 (0.725–0.961) *	0.758 (0.541–1.061)	
Smoking status				0.049
Current smoker	1.0	0.901 (0.731–1.112)	0.876 (0.621–1.236)	
Never smoker	1.0	0.805 (0.732–0.886) ***	0.851 (0.772–0.938) **	
Alcohol consumption				0.267
Nondrinkers	1.0	0.825 (0.728–0.935) **	0.854 (0.652–1.118)	
Low-moderate	1.0	0.816 (0.715–0.930) **	0.947 (0.744–1.204)	
Heavy	1.0	0.866 (0.625–1.200)	0.564 (0.335–0.949) *	
Average sleep time (hours/day)				0.106
<6	1.0	0.765 (0.666–0.879) ***	0.731 (0.558–0.957) *	
6–6.9	1.0	0.870 (0.788–0.973) *	0.955 (0.768–1.187)	
BMI (kg/m^2^)				<0.001
Normal (18.5~24.9)	1.0	0.764 (0.637–0.915) **	0.644 (0.427–0.972) *	
Obesity (≥25.0)	1.0	0.636 (0.542–0.747) ***	0.719 (0.538–0.961) *	

OR, odds ratio; CI, confidence interval; PA, physical activity. * *p* < 0.05, ** *p* < 0.01, *** *p* < 0.001.

**Table 6:** Subgroup analysis of diabetes according to physical activity group among adults with sleep deficiency.

Subgroup	Low PA OR (95% CI)	Moderate PA OR (95% CI)	High PA OR (95% CI)	*p* for Interaction
Sex				0.156
Men	1.0	0.967 (0.847–1.105)	0.746 (0.595–0.936) *	
Women	1.0	0.873 (0.775–0.984) *	0.704 (0.603–0.821) *	
Age (years)				0.264
20~39	1.0	1.085 (0.855–1.377)	1.019 (0.702–1.477)	
40~59	1.0	0.731 (0.631–0.846) **	0.596 (0.460–0.773) ***	
60~64	1.0	0.923 (0.801–1.063)	0.818 (0.586–1.142)	
Smoking status				0.748
Current smoker	1.0	0.941 (0.757–1.168)	0.785 (0.545–1.131)	
Never smoker	1.0	0.905 (0.822–0.997) *	0.729 (0.594–0.894) **	
Alcohol consumption				0.757
Nondrinkers	1.0	0.976 (0.860–1.107)	0.753 (0.567–0.999) *	
Low-moderate	1.0	0.860 (0.753–0.983) *	0.769 (0.596–0.991) *	
Heavy	1.0	0.810 (0.565–1.160)	0.607 (0.353–1.043)	
Average sleep time (hours/day)				0.437
<6	1.0	0.768 (0.613–0.963) *	0.690 (0.522–0.913) **	
6–6.9	1.0	0.913 (0.815–1.022)	0.777 (0.618–0.978) *	
BMI (kg/m^2^)				<0.001
Normal (18.5~24.9)	1.0	0.910 (0.805–1.027)	0.845 (0.661–1.081)	
Obesity (≥25.0)	1.0	0.868 (0.756–0.997) *	0.623 (0.477–0.813) **	

OR, odds ratio; CI, confidence interval; PA, physical activity. * *p* < 0.05, ** *p* < 0.01, *** *p* < 0.001.

**Table 7:** Subgroup analysis of dyslipidemia according to physical activity group among adults with sleep deficiency.

Subgroup	Low PA OR (95% CI)	Moderate PA OR (95% CI)	High PA OR (95% CI)	*p* for Interaction
Sex				0.367
Men	1.0	1.026 (0.903–1.165)	0.827 (0.669–1.024)	
Women	1.0	0.987 (0.877–1.109)	1.008 (0.768–1.324)	
Age (years)				0.080
20~39	1.0	0.898 (0.732–1.103)	0.778 (0.558–1.086)	
40~59	1.0	0.948 (0.834–1.077)	0.934 (0.731–1.193)	
60~64	1.0	1.141 (0.988–1.318)	0.871 (0.624–1.217)	
Smoking status				0.207
Current smoker	1.0	1.024 (0.831–1.262)	0.692 (0.491–0.976) *	
Never smoker	1.0	0.996 (0.907–1.093)	0.963 (0.796–1.165)	
Alcohol consumption				0.626
Nondrinkers	1.0	1.062 (0.939–1.201)	1.068 (0.821–1.390)	
Low-moderate	1.0	0.926 (0.816–1.052)	0.800 (0.630–1.016)	
Heavy	1.0	1.079 (0.886–1.786)	1.041 (0.609–1.780)	
Average sleep time (hours/day)				0.375
<6	1.0	0.921 (0.802–1.057)	0.933 (0.717–1.215)	
6–6.9	1.0	1.051 (0.944–1.170)	0.870 (0.702–1.078)	
BMI (kg/m^2^)				< 0.001
Normal (18.5~24.9)	1.0	1.001 (0.892–1.122)	1.017 (0.809–1.278)	
Obesity (≥25.0)	1.0	0.990 (0.861–1.137)	0.729 (0.563–0.944) *	

OR, odds ratio; CI, confidence interval; PA, physical activity. * *p* < 0.05.

**Table 8:** Subgroup analysis of hypertension according to physical activity group among adults with sleep deficiency.

Subgroup	Low PA OR (95% CI)	Moderate PA OR (95% CI)	High PA OR (95% CI)	*p* for Interaction
Sex				0.038
Men	1.0	0.953 (0.835–1.088)	0.747 (0.598–0.935) *	
Women	1.0	0.890 (0.785–1.010)	0.857 (0.634–1.160)	
Age (years)				0.186
20~39	1.0	0.997 (0.784–1.267)	1.068 (0.740–1.540)	
40~59	1.0	0.941 (0.823–1.074)	0.690 (0.532–0.894) **	
60~64	1.0	0.930 (0.802–1.079)	0.794 (0.563–1.120)	
Smoking status				0.123
Current smoker	1.0	1.014 (0.814–1.264)	0.788 (0.544–1.142)	
Never smoker	1.0	0.904 (0.818–0.999) *	0.785 (0.638–0.966) *	
Alcohol consumption				0.046
Nondrinkers	1.0	0.918 (0.806–1.047)	0.661 (0.492–0.888) **	
Low-moderate	1.0	0.974 (0.851–1.116)	0.961 (0.746–1.239)	
Heavy	1.0	0.703 (0.495–0.998) *	0.535 (0.313–0.914) *	
Average sleep time (hours/day)				0.743
<6	1.0	0.835 (0.721–0.966) *	0.700 (0.527–0.930) *	
6–6.9	1.0	0.983 (0.876–1.103)	0.847 (0.671–1.070)	
BMI (kg/m^2^)				<0.001
Normal (18.5~24.9)	1.0	0.963 (0.852–1.089)	0.735 (0.569–0.949) *	
Obesity (≥25.0)	1.0	0.871 (0.753–0.992) *	0.732 (0.612–0.875) **	

OR, odds ratio; CI, confidence interval; PA, physical activity. * *p* < 0.05, ** *p* < 0.01.

**Table 9:** Subgroup analysis of metabolic syndrome according to physical activity group among adults with sleep deficiency.

Subgroup	Low PA OR (95% CI)	Moderate PA OR (95% CI)	High PA OR (95% CI)	*p* for Interaction
Sex				0.003
Men	1.0	0.933 (0.817–1.064)	0.656 (0.520–0.828) ***	
Women	1.0	0.814 (0.716–0.925) **	0.778 (0.681–0.889) ***	
Age (years)				0.497
20~39	1.0	0.892 (0.692–1.149)	0.814 (0.543–1.221)	
40~59	1.0	0.830 (0.722–0.954) **	0.638 (0.486–0.839) ***	
60~64	1.0	0.935 (0.812–1.076)	0.714 (0.512–0.996) *	
Smoking status				0.271
Current smoker	1.0	0.919 (0.740–1.142)	0.589 (0.401–0.865) **	
Never smoker	1.0	0.857 (0.776–0.948) **	0.740 (0.598–0.917) **	
Alcohol consumption				0.471
Nondrinkers	1.0	0.917 (0.806–1.043)	0.693 (0.516–0.931) *	
Low-moderate	1.0	0.817 (0.711–0.940) **	0.783 (0.598–1.024)	
Heavy	1.0	0.855 (0.609–1.199)	0.483 (0.282–0.829) **	
Average sleep time (hours/day)				0.037
<6	1.0	0.738 (0.639–0.852) ***	0.649 (0.487–0.864) **	
6–6.9	1.0	0.961 (0.855–1.080)	0.738 (0.578–0.943) *	
BMI (kg/m^2^)				< 0.001
Normal (18.5~24.9)	1.0	0.891 (0.772–1.027)	0.712 (0.520–0.974) *	
Obesity (≥25.0)	1.0	0.781 (0.677–0.903) ***	0.608 (0.464–0.795) ***	

OR, odds ratio; CI, confidence interval; PA, physical activity. * *p* < 0.05, ** *p* < 0.01, *** *p* < 0.001.

**Table 10:** Subgroup analysis of depression according to physical activity group among adults with sleep deficiency.

Subgroup	Low PA OR (95% CI)	Moderate PA OR (95% CI)	High PA OR (95% CI)	*p* for Interaction
Sex				0.715
Men	1.0	1.246 (0.838–1.851)	1.799 (1.009–32,082) *	
Women	1.0	1.052 (0.848–1.305)	1.550 (1.004–2.394) *	
Age (years)				0.384
20~39	1.0	0.928 (0.578–1.489)	2.266 (1.230–4.175) **	
40~59	1.0	1.026 (0.756–1.393)	1.157 (0.647–2.069)	
60~64	1.0	1.236 (0.935–1.634)	1.684 (0.877–3.237)	
Smoking status				0.328
Current smoker	1.0	1.170 (0.693–1.975)	2.963 (1.458–6.021) **	
Never smoker	1.0	1.075 (0.878–1.316)	1.382 (0.922–2.071)	
Alcohol consumption				0.722
Nondrinkers	1.0	1.152 (0.902–1.471)	1.778 (1.104–2.664) *	
Low-moderate	1.0	1.082 (0.790–1.483)	1.544 (0.897–2.657)	
Heavy	1.0	0.548 (0.212–1.414)	1.250 (0.332–4.709)	
Average sleep time (hours/day)				0.002
<6	1.0	1.248 (0.953–1.634)	1.747 (1.074–2.842) *	
6–6.9	1.0	0.959 (0.735–1.252)	1.463 (0.895–2.392)	
BMI (kg/m^2^)				0.133
Normal (18.5~24.9)	1.0	1.004 (0.784–1.285)	1.784 (1.166–2.729) **	
Obesity (≥25.0)	1.0	1.274 (0.939–1.727)	1.197 (0.570–2.059)	

OR, odds ratio; CI, confidence interval; PA, physical activity. * *p* < 0.05, ** *p* < 0.01.

## Data Availability

The KNHANES data used in this study are available at https://knhanes.kdca.go.kr/knhanes/main.do (accessed on 10 May 2025).

## Supplementary Materials

The following supporting information can be downloaded at: https://www.mdpi.com/article/10.3390/jcm14238398/s1, File S1. Anthropometric measurements, blood pressure, and biochemical assessments.
