# Supplementary material for: Association Between Physical Activity Levels and Chronic Disease Risk Among Korean Adults with Sleep Deficiency
Source: J Clin Med. 2025 Nov 26;14(23):8398. doi: 10.3390/jcm14238398 (PMC12692821; doi:10.3390/jcm14238398)
Supplement: Supplementary file 1 [file jcm-14-08398-s001.zip › jcm-3989052-supplementary.pdf]

Supplementary method

S1. Anthropometric measurements, blood pressure, and biochemical assessments

Participants' height was measured using a stadiometer (Seca 225, Seca, Hamburg, Germany) while standing barefoot. Body weight was assessed using a digital scale (GL-6000-20, G-tech, Seoul, Republic of Korea). Body mass index (BMI) was calculated as the weight in kilograms divided by the height in meters squared (kg/m^2). Waist circumference (WC) was measured at the midpoint between the lower margin of the last rib and the superior border of the iliac crest using non-stretchable tape (Seca 200, Seca, Hamburg, Germany). Blood pressure (BP) was measured using a mercury sphygmomanometer (Wall Unit 33, Baumanometer, NY, USA), and both systolic and diastolic pressures were recorded.

Venous blood samples were collected after at least an 8-hour fasting period. Total cholesterol (TC), triglycerides (TG), low-density lipoprotein cholesterol (LDL-C), high-density lipoprotein cholesterol (HDL-C), insulin, and fasting blood glucose (FBG) levels were measured using enzymatic colorimetric methods with an automated analyzer (Hitachi Automatic Analyzer 7600-210, Hitachi Medical Corp., Tokyo, Japan). Glycated hemoglobin (HbA1c) levels were determined by high-performance liquid chromatography (HPLC) using a dedicated analyzer (Tosoh G8; Tosoh Corp., Tokyo, Japan). Aspartate aminotransferase (AST) and alanine aminotransferase (ALT) levels were measured using the IFCC UV method without pyridoxal-5'-phosphate (P5P) on the same automated analyzer (Hitachi Automatic Analyzer 7600-210, Hitachi Medical Corp., Tokyo, Japan). The homeostasis model assessment of insulin resistance (HOMA-IR) was calculated using the following formula (fasting insulin [μU/mL] * fasting glucose [mg/dL] / 405) [10].
